# Supplementary material for: Dynalign II: common secondary structure prediction for RNA homologs with domain insertions
Source: Nucleic Acids Res. 2014 Nov 21;42(22):13939–48. doi: 10.1093/nar/gku1172 (PMC4267632; doi:10.1093/nar/gku1172)
Supplement: SUPPLEMENTARY DATA [file supp_gku1172_nar-02021-z-2014-File012.zip › manual/javadoc/RNAstructure_java_utilities/SimpleDialogHandler.html]

SimpleDialogHandler


JavaScript is disabled on your browser.


- Overview
- Package
- Class
- Tree
- Index
- Help

- Prev Class
- Next Class

- Frames
- No Frames

- All Classes

- Summary:
- Nested |
- Field |
- Constr |
- Method

- Detail:
- Field |
- Constr |
- Method


RNAstructure\_java\_utilities

## Class SimpleDialogHandler

- java.lang.Object
- - RNAstructure\_java\_utilities.SimpleDialogHandler

- All Implemented Interfaces:
  :   Serializable

  ---

    

  ```
  public class SimpleDialogHandler
  extends Object
  implements Serializable
  ```

  A class that handles simple dialogs used in the RNAstructure GUI.

  See Also:
  :   Serialized Form

- - ### Field Summary

    Fields

    | Modifier and Type | Field and Description |
    | --- | --- |
    | `private static long` | `serialVersionUID` |
  - ### Constructor Summary

    Constructors

    Constructor and Description

    `SimpleDialogHandler()`
  - ### Method Summary

    Methods

    | Modifier and Type | Method and Description |
    | --- | --- |
    | `void` | `makeErrorDialog(String message)` Show an error dialog. |
    | `Double` | `makeInputDialog(NumberField.DoubleField field)` Show an input dialog that gets a double within a certain range. |
    | `Integer` | `makeInputDialog(NumberField.IntegerField field)` Show an input dialog that gets an integer within a certain range. |
    | `void` | `makeMessageDialog(Object message)` Show a message dialog. |
    | `void` | `makeMessageDialog(String msg)` Show a message dialog. |
    | `String` | `makeThreeChoicesDialog(Object message)` Show a dialog that gives the user one of three choices to make. |
    | `String` | `makeThreeChoicesDialog(String msg)` Show a dialog that gives the user one of three choices to make. |
    | `String` | `makeTwoChoicesDialog(Object message)` Show a dialog that gives the user one of two choices to make. |
    | `String` | `makeTwoChoicesDialog(String msg)` Show a dialog that gives the user one of two choices to make. |
    | `void` | `makeWarningDialog(String message)` Show a warning dialog. |

    - ### Methods inherited from class java.lang.Object

      `clone, equals, finalize, getClass, hashCode, notify, notifyAll, toString, wait, wait, wait`

- - ### Field Detail


    - #### serialVersionUID

      ```
      private static final long serialVersionUID
      ```

      See Also:
      :   Constant Field Values
  - ### Constructor Detail


    - #### SimpleDialogHandler

      ```
      public SimpleDialogHandler()
      ```
  - ### Method Detail


    - #### makeErrorDialog

      ```
      public void makeErrorDialog(String message)
      ```

      Show an error dialog.

      Parameters:
      :   `message` - The message on the dialog.


    - #### makeInputDialog

      ```
      public Double makeInputDialog(NumberField.DoubleField field)
      ```

      Show an input dialog that gets a double within a certain range.

      Parameters:
      :   `field` - The double input field.

      Returns:
      :   The double selected from this field.


    - #### makeInputDialog

      ```
      public Integer makeInputDialog(NumberField.IntegerField field)
      ```

      Show an input dialog that gets an integer within a certain range.

      Parameters:
      :   `field` - The integer input field.

      Returns:
      :   The integer selected from this field.


    - #### makeMessageDialog

      ```
      public void makeMessageDialog(Object message)
      ```

      Show a message dialog.

      Parameters:
      :   `message` - The message on the dialog.


    - #### makeMessageDialog

      ```
      public void makeMessageDialog(String msg)
      ```

      Show a message dialog.

      Parameters:
      :   `msg` - The message on the dialog.


    - #### makeThreeChoicesDialog

      ```
      public String makeThreeChoicesDialog(Object message)
      ```

      Show a dialog that gives the user one of three choices to make.

      Parameters:
      :   `message` - The message on the dialog.

      Returns:
      :   The response: "YES", "NO", or "CANCEL".


    - #### makeThreeChoicesDialog

      ```
      public String makeThreeChoicesDialog(String msg)
      ```

      Show a dialog that gives the user one of three choices to make.

      Parameters:
      :   `msg` - The message on the dialog.

      Returns:
      :   The response: "YES", "NO", or "CANCEL".


    - #### makeTwoChoicesDialog

      ```
      public String makeTwoChoicesDialog(Object message)
      ```

      Show a dialog that gives the user one of two choices to make.

      Parameters:
      :   `message` - The message on the dialog.

      Returns:
      :   The response: "OK" or "CANCEL".


    - #### makeTwoChoicesDialog

      ```
      public String makeTwoChoicesDialog(String msg)
      ```

      Show a dialog that gives the user one of two choices to make.

      Parameters:
      :   `msg` - The message on the dialog.

      Returns:
      :   The response: "OK" or "CANCEL".


    - #### makeWarningDialog

      ```
      public void makeWarningDialog(String message)
      ```

      Show a warning dialog.

      Parameters:
      :   `message` - The message on the dialog.


- Overview
- Package
- Class
- Tree
- Index
- Help

- Prev Class
- Next Class

- Frames
- No Frames

- All Classes

- Summary:
- Nested |
- Field |
- Constr |
- Method

- Detail:
- Field |
- Constr |
- Method
